# Shelf acetabuloplasty is associated with longer survival of the total hip arthroplasty in patients with late-detected hip dislocation: a cohort study of 70 hips with a modified Spitzy shelf procedure

**DOI:** 10.2340/17453674.2025.44036

**Published:** 2025-07-07

**Authors:** Terje TERJESEN, Stefan HUHNSTOCK

**Affiliations:** Section of Children’s Orthopaedics and Reconstructive Surgery, Division of Orthopaedic Surgery, Oslo University Hospital, Rikshospitalet, and University of Oslo, Oslo, Norway

## Abstract

**Background and purpose:**

Our aim was to compare age at time of total hip arthroplasty (THA) and the THA survival time in patients with late-detected developmental dislocation and dysplasia of the hip (DDH) treated with traction in childhood who had either undergone acetabular shelf operation due to persistent DDH, or no previous acetabular surgery but persistent DDH, or no previous acetabular surgery and CE angle ≥ 18°.

**Methods:**

112 patients (97 females; 144 hips) with late-detected DDH who had undergone THA were studied. 70 hips had undergone a modified Spitzy procedure (SA group) at the age of 8–33 years. They were compared with 2 groups that had not undergone previous pelvic surgery: a “Dysplasia” group with residual (persistent) acetabular dysplasia (CE angle < 18°, 33 hips) and a “Normal” group with no residual dysplasia (37 hips). We analyzed age at THA and the survival rate (percentage of THAs not having undergone revision).

**Results:**

Mean patient age at THA did not differ between the SA group (52 years) and the Dysplasia group (49 years; P = 0.1). 11 THAs had been revised in the SA group and 9 in the Dysplasia group. Kaplan–Meier analysis showed 20-year survival rates of 88% in the SA group and 68% in the Dysplasia group. The estimated survival time of THA was significantly higher in the SA group than in the Dysplasia group (29.4 and 19.8 years; P = 0.01). Mean age at THA was significantly lower in the Dysplasia group than in the Normal group (49 and 55 years), but there was no significant difference between these groups in estimated survival time of THA.

**Conclusion:**

A previous acetabular shelf operation in patients with persistent DDH does not appear to delay age at THA but THA had better survival rate.

Congenital dislocation and developmental dysplasia of the hip (DDH) are among the most common causes of secondary hip osteoarthritis (OA). Acetabular dysplasia with or without subluxation after initial treatment is a risk factor for later OA [1-3]. Joint-preserving surgical procedures like periacetabular osteotomy (PAO) and acetabular shelf operation (shelf acetabuloplasty, SA) are widely used to correct residual (persistent) acetabular dysplasia with low femoral head coverage (FHC) [4-7].

Shelf acetabuloplasty is the oldest surgical procedure to increase FHC in patients with residual DDH [8]. It provides an extended acetabular roof by inserting a corticocancellous autograft at the superior antero-lateral rim of the dysplastic acetabulum. This may ensure improved support to the femoral head and a more stable joint. The intentions are to relieve pain and improve function in the short term [9,10]. In the longer term, it has been claimed to postpone the occurrence of OA and the need for total hip arthroplasty (THA) [7,11,12].

The increased bone stock in the acetabulum could be beneficial if a later THA is needed [6,13,14]. The shelf would facilitate the surgical procedure as bone grafting at the acetabular side may not be required. Furthermore, it is possible that the buttress of bone could provide better fixation of the acetabular component and thus prolong the survival of the THA, but this was not confirmed by Benad et al. [15].

The aim of our study was to compare age at THA and the THA survival in patients with late-detected DDH treated with traction in childhood who had either (i) undergone acetabular shelf operation due to persistent DDH (CE angle < 18°), or (ii) had no previous acetabular surgery but persistent DDH, or (iii) no previous acetabular surgery and a CE angle ≥ 18°.

## Methods

### Study design

The study is reported according to STROBE guidelines for observational studies.

Patients with late-detected DDH were identified from 3 sources at Sophies Minde Orthopaedic Hospital (SMOH, now the Division of Orthopaedic Surgery, Oslo University Hospital).

### Inclusion criteria

48 patients without SA had been identified by a search of our diagnosis card index for the period 1958–1962 [16]. 44 patients who had undergone Spitzy SA were identified by a search of surgical procedures for the period 1954–1976 [17]. Because the number of patients from these sources was too small for a study on survival of THA, a search of the radiographic files at SMOH was performed, identifying 164 “new” patients with late-detected DDH, treated during the period 1946–1980. Thus, 256 patients were identified.

**Figure 1:**
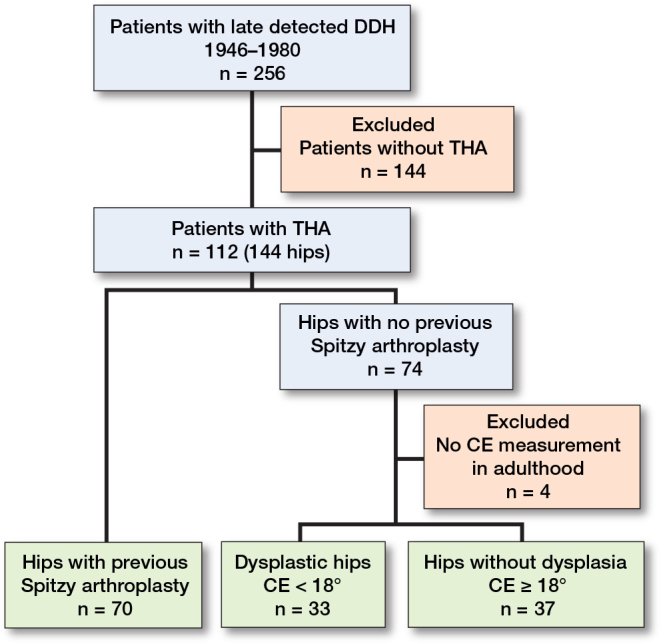
Patient flowchart.

The inclusion criteria were no associated anomaly, age at diagnosis ≥ 3 months, initial treatment with skin traction to obtain closed reduction, and THA because of OA in adulthood. 112 patients (144 hips) fulfilled these criteria. An overview of the patients is shown in Figure 1. Several patients had during childhood and early adulthood undergone femoral varus and/or derotation osteotomy, which we did not consider to be associated with either the age at, or survival of, a later THA.

### Patient groups

The patients were divided into 3 groups according to previous shelf acetabuloplasty (SA) and the degree of residual acetabular dysplasia.

**Figure 2:**
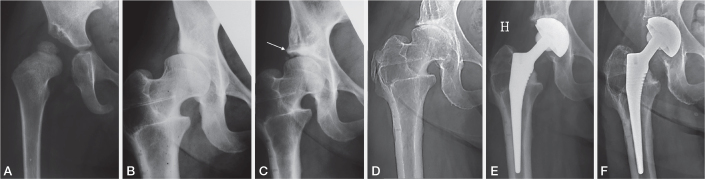
(A) Initial pelvic radiograph of a girl at the age of 2.7 years, showing a dislocated right hip. (B) The same patient at the age of 20 years shows residual dysplasia of the right hip. A varus femoral osteotomy had been performed at the age of 16 years. A modified Spitzy shelf arthroplasty was performed 10 months after this radiograph. (C) Radiograph 3 years after the Spitzy operation, showing improvement of the acetabular roof and satisfactory femoral head coverage. The shelf is indicated by an arrow. (D) Radiograph at the age of 48 years, showing osteoarthritis of the right hip with narrowing of the joint space. (E) Radiograph 3 months after total hip arthroplasty. (F) Last radiograph at patient age 69 years, showing no signs of loosening of the hip prosthesis.

*Group 1.* The SA group consisted of 59 patients (70 hips), 53 females and 6 males, with a mean age of 16.0 years (8–33) at the time of SA. Indication for SA was residual acetabular dysplasia defined as a center–edge (CE) angle < 20° [1], with or without hip pain (Figure 2). The surgical technique was a modified Spitzy procedure as described by Holm et al. [12]. There was no hip joint OA at the time of SA.

53 patients (74 hips), 44 females and 9 males, had been treated for late-detected DDH in childhood with initial skin traction to obtain closed reduction, but had not undergone any pelvic operations (osteotomy or SA), either because the hips had become normal after the initial treatment or they had residual dysplasia, but no hip pain. These hips were divided into 2 groups according to CE angle 18°, which was the highest preoperative CE angle in the SA group. Radiographs at skeletal maturity were not available in 4 hips. There were no significant differences between individuals with and without SA regarding distribution of sex or age < 50 years vs ≥ 50 years at the time of THA.

*Group 2.* 33 hips had residual (persisting) acetabular dysplasia in adulthood with CE angle < 18° (Dysplasia group).

*Group 3.* 37 hips had no or very mild dysplasia in adulthood with CE angle ≥ 18° (Normal group).

Radiographs taken in adulthood of almost all the patients were available for evaluation. The CE angle was measured at the last preoperative radiograph and 1 year postoperatively in the SA group and in adulthood (age > 15 years) in the individuals without SA. The radiographic assessment was performed by 1 of the authors (TT), who has long experience with evaluation of radiographs of hips in children and adults.

### Total hip arthroplasty

Information regarding THA was obtained from the Norwegian Arthroplasty Register (NAR), which started its registration in 1988. The data was received in May 2024 and gave information on the date of THA, whether bone cement had been used, the date of revision of the THA if this had been performed, and the reasons for revision. The age at THA and the survival rate of the THA were compared between the 3 groups. The survival rate was defined as the percentage of hips that had not undergone revision according to time after THA. Survival time was defined as the time from THA to revision in hips that had undergone revision and to May 2024 for hips that had not undergone revision, and from THA to the time of death in patients who had died during the follow-up period.

### Statistics

SPSS (version 29) was used for statistical analysis (IBM Corp, Armonk, NY, USA). Categorical data was analyzed with the Pearson chi-square test. Continuous variables were tested for normal distribution with the Kolmogorov–Smirnov test. Variables with normal distribution were analyzed using the t-test for independent samples. For correlation between parameters, Pearson’s correlation coefficient (r) was used. All tests were 2-sided. Differences were considered significant when the P value was < 0.05. Kaplan–Meier survival analysis was used to assess the survival rate of THA with time, and the log-rank test was used to compare the estimated survival time between groups of hips.

### Ethics, funding, and disclosures

The study was approved by the Regional Committee of Medical Research Ethics (ref. 25480) and our institutional review board, and informed consent was received from all the patients.

The study did not receive financial funding. There are no conflicts of interest. Complete disclosure of interest forms according to ICMJE are available on the article page, doi: 10.2340/17453674.2025.44036

## Results

### Patient characteristics

The mean preoperative CE angle in the SA group was 6.8° (range –20 to 18) and the mean CE angle 1 year postoperatively was 32° (range 4–47). In the other groups, the mean CE angle was 12° (range 3–17) at skeletal maturity in the Dysplasia group and 24° (range 18–35) in the Normal group (Table 1).

**Table 1:** Comparison between hips with Spitzy acetabuloplasty and the 2 groups without previous pelvic surgery

Variables	SA group n = 70	Dysplasia group CE < 18°n = 33	Normal group CE ≥ 18°n = 37	Comparison		
				P ^a^	P ^b^	P ^c^
CE angle °, mean (SD)	6.8 (8.2)	12 (4.5)	24 (4.3)	0.001	< 0.001	< 0.001
Age at THA ^d^, mean (SD)	52 (7.8)	49 (10)	55 (7.5)	0.1	0.06	0.005
Estimated survival time of THA, years, mean (CI) ^e^	29.4 (26.3–32.6)	19.8 (16.7–28.9)	26.6 (21.8–31.3)	0.01	0.4	0.3

SA: Spitzy acetabuloplasty; CE: center edge, in the SA group it is the CE angle before SA; THA: total hip arthroplasty.

a Comparison between the SA group and the Dysplasia group (CE angle < 18°).

b Comparison between the SA group and the Normal group (CE angle ≥ 18°).

c Comparison between the Dysplasia group and the Normal group.

d t-test for independent samples.

e Kaplan–Meier survival analysis with log-rank test; CI: 95% confidence interval.

The mean patient age at THA was 52 years (range 37–71) in the SA group, 49 (range 33–65) in the Dysplasia group and 55 years (range 39–71) in the Normal group (Table 1). The differences between the SA group and the other groups were not statistically significant. The age at THA was significantly lower in the Dysplasia group than in the Normal group (P = 0.005). In the SA group, there was no significant correlation between age at THA and preoperative CE angle or CE angle 1 year after SA. In hips that had not undergone SA, there was a significant correlation between the CE angle at skeletal maturity and age at THA (r = 0.41; P < 0.001).

### Total hip arthroplasty

The THAs had been performed during the period 1988–2023 (median 2003) in the SA group and during 1986–2022 (median 2011) in hips with no SA (P = 0.001). During follow-up, 8 patients in the SA group and 1 in the Normal group died. As none of their THAs had been revised, they were included in the follow-up analysis but censored at the time of death.

25 THAs had been revised, 11 in the SA group (16%), 9 in the Dysplasia group (27%), and 5 in the Normal group (14%). There were no statistically significant differences in reasons for revision between the 3 groups (Table 2). Reasons for revision of THA were loose acetabular cup (11 hips, worn-out cup (2 hips), fracture of the cup liner (2 hips), loose femoral stem (1 hip), osteolysis in femur (2 hips, femoral fracture (1 hip), THA dislocation (3 hips), and pain in the hip region (3 hips). The reasons for revision were acetabular-related (loose cup, worn-out cup or fracture of the liner) in 7 of 11 hips in the SA group, in 7 of 9 hips in the Dysplasia group, and in 1 of 5 hips in the Normal group. The Dysplasia group had significantly more acetabular-related revisions than the normal group (P = 0.04), but there was no differences between the SA group and the Dysplasia group (P = 0.5) or the Normal group (P = 0.1).

**Table 2:** Reasons for revision of total hip arthroplasty according to patient groups

Reasons for THA revision	SA group n = 70	Dysplasia group CE < 18° n = 33	Normal group CE ≥ 18° n = 37	Comparison		
				P ^a^	P ^b^	P ^c^
Loose acetabular cup	4	6	1	0.5	0.4	0.3
Worn-out cup	1	1	0			
Fracture of cup liner	2	0	0			
Loose femoral stem	0	0	1			
Osteolysis in femur	2	0	0			
Femoral fracture	0	0	1			
THA dislocation	1	1	1			
Pain in hip region	1	1	1			
Total	11	9	5			

For ^a–c^ and abbreviations, see Table 1.

**Figure 3:**
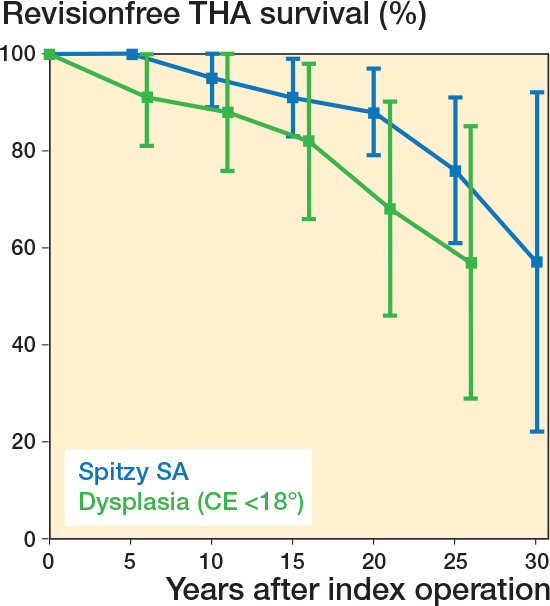
Kaplan–Meier survival analysis (with 95% confidence intervals) of the THA, showing percentage of patients not having undergone revision of the arthroplasty. The modified Spitzy SA group is shown by a blue line and the Dysplasia group by a green line.

The survival rate of THA in the SA group was 95% at 10 years, decreasing to 88% at 20 years and 57% at 30 years (Tables 3; Figure 3). In the Dysplasia group, the 10-year survival rate was 88% and 20-year survival was 68%. The estimated mean survival time (Kaplan–Meier analysis and log-rank test) was 29.4 years in the SA group and 19.8 years in the Dysplasia group (Table 1); the difference was statistically significant (P = 0.01). The estimated mean survival time in the Normal group was 26.6 years (95% confidence interval [CI] 21.8–31.3), which was not significantly different from the other 2 groups.

**Table 3:** Kaplan–Meier survival analysis of THA in hip dysplasia patients, with revision of THA as endpoint, in the group with Spitzy acetabuloplasty (SA group) and the groups without SA

Time, years	SA group n = 70			Dysplasia group (CE < 18°) n = 33			Normal group (CE ≥ 18°) n = 37		
	Events	Survival	Remaining	Events	Survival	Remaining	Events	Survival	Remaining
5	0	100	62	3	91 (81–100	29	2	94 (85–100)	28
10	3	95 (89–100)	51	4	88 (76–100)	20	4	86 (73–99)	18
15	6	88 (79–97)	41	5	82 (66–98)	12	4	86 (73–99)	8
20	6	88 (79–97)	28	7	68 (46–90)	6	5	74 (49–99)	4
25	9	76 (61–91)	13	9	57 (29-85)	0	5	74 (49–99)	0
30	10	57 (22–92)	3						

THA: total hip arthroplasty; Time: years after THA; Events: number of revised THAs; Survival: percentage (with 95% confidence interval) of THA not revised; Remaining: number of THAs not revised.

Fixation without bone cement had been used in 107 acetabular components (77%), bone cement had been used in 32 components, and there was missing information in 5. Bone cement had been used in 22 femoral components, had not been used in 118 components (84%), and there was missing information in 4. There was no significant difference in the use of uncemented components between the SA group and the other groups. Neither was there any significant association between the proportion of uncemented/cemented components and whether or not the THA components had been revised.

## Discussion

The aim of our study was to compare age at THA and the THA survival time in patients with late-detected DDH treated with traction in childhood who had either undergone acetabular shelf operation due to persistent DDH (CE angle < 18°), or no previous acetabular surgery but persistent DDH, or no previous acetabular surgery and a CE angle ≥ 18°.

We showed that patients who had undergone an acetabular shelf operation because of persistent DDH (CE angle < 18°) had significantly longer survival time of a later THA compared with patients with persistent DDH but no previous acetabular surgery, whereas there was no significant difference in age at THA. In patients without previous pelvic surgery, the age at THA was significantly lower in patients with persistent DDH than in patients without persistent dysplasia, but there was no significant difference in the survival of the THA.

One intention of shelf acetabuloplasty has been to delay the occurrence of OA and the need for THA in the long term [7,11,12]. However, this has not been clarified, mainly because no randomized studies with a control group exist. A randomized study would be almost impossible to perform because one of the reasons for performing SA is hip pain, and a symptomatic patient would hardly accept to be randomized to a control group with no surgery. The control group of individuals with DDH and THA but no previous pelvic operation should preferably be composed of patients with the same degree of DDH as the study group. Thus, our Dysplasia group, with CE angle < 18°, was considered to be an adequate control group, because the highest preoperative CE angle in the SA group was 18°.

We found no significant difference in age at THA between the SA group (52 years) and the Dysplasia group (49 years), which is in accordance with the results of Benad et al. (47 and 49 years, respectively) [15]. The long-term outcome of PAO showed that approximately 50% of the hips had undergone THA or had had progression of OA at patient age 50 years [18], which is in keeping with the survival rate of SA at the same age [17]. The mean age at THA in other studies of hips with DDH and no prior pelvic procedures varied between 43 and 51 years (Table 4), which is considerably younger compared with the mean age at THA of 70 years in individuals with idiopathic OA [19].

**Table 4:** Age at THA and survival of THA in patients with DDH according to follow-up time after surgery in the present study and in recent previous studies

Authors	Previous pelvic procedure	Hips, n	Age at THA	THA survival (%) at				
				5	10	15	20	25 years
Chougle (2006)	No	271	43		91		70	
Janz (2021)	No	72	51		97		86	
Benad (2022)	No	63	49			83		
Fahlbusch (2023)	No	96	51		98		84	
Present study (2025) ^a^	No	74	52	91	88	82	74	
Benad (2022)	Shelf acetabuloplasty	61	47			89		
Present study (2025)	Spitzy shelf acetabuloplasty	70	52	100	95	88	88	76

THA: Total hip arthroplasty; DDH: developmental dislocation and dysplasia of the hip.

a All hips with no previous pelvic surgery.

The increased bone stock in the lateral part of the dysplastic acetabulum provided by SA can facilitate a later THA, as an extra bone graft to augment the acetabulum is not required [6,14,21,22]. Theoretically, the increased bone stock should give better fixation of the cup and lead to longer survival of the THA, but this has so far not been clarified. In the only previous study with a control group, the 15-year survival rate of the THA was 89% in the SA group and 83% in the control group (P = 0.6) [15]. These results were almost identical to ours, where the 15-year survival rates were 88% and 82%, respectively. Other studies of patients with DDH but no prior pelvic procedures showed 20-year survival rates of 70–86% [22-24], which is in accordance with the rate of 74% in our study (Table 4). Fahlbusch et al. [24] reported a significantly higher survival rate of THA at 20 years in hips without prior pelvic surgery (87%) compared with hips with prior pelvic osteotomy (64%). In a study of THA with cemented acetabular components [22], the 20-year acetabular revision rate was 23% in hips without previous acetabular surgery and 70% in hips with previous surgery. This high revision rate differs markedly from ours; however, we used a modified Spitzy technique while various procedures (femoral osteotomy, shelf procedure, Chiari osteotomy) had been used in the study of Chougle et al. [22]. The reasons for the differences could be differences in degrees of DDH and surgical procedures. Yacovelli et al. [25] pointed out that THA after a prior pelvic osteotomy is technically demanding, leading to longer operative time, greater blood loss, and variation in implant placement.

We divided the non-SA patients into 2 groups because this made it possible to evaluate the effects of different degrees of residual dysplasia. The mean age at THA was higher in the Normal group than in the Dysplasia group (55 and 49 years, respectively). There was a significant relationship between the CE angle and the age at THA, which is in accordance with Wiberg [1] who found a linear relationship between the CE angle and the age at onset of OA. No significant difference was found in the survival time of the THAs between the 2 groups. This indicated that persistent acetabular dysplasia is associated with lower age at THA but not with time to revision of the THA. However, the latter conclusion must be viewed with caution as the number of revisions was rather small.

The reasons for revision were related to the cup in 7 of the 9 hips in the Dysplasia group and in 1 of 5 hips in the Normal group. Although the numbers are too small to draw definite conclusions, this difference could be associated with the amount of bone in the acetabular roof.

### Limitations

First, the study was retrospective, and there was no randomization of the patients. Thus, although the SA group and the Dysplasia group had similar degrees of acetabular dysplasia, they were somewhat unequal, because patients in the SA group usually had clinical symptoms, whereas patients in the Dysplasia group probably had no or minor symptoms during adolescence and young adulthood. Second, the follow-up period after THA was relatively short in many patients and the number of revised THAs was rather small, which could affect the reliability of the statistical analyses. Third, the effect of potential confounding factors like the type of THA was not evaluated because of too few patients according to each type of THA. Likewise, the effect of the use of bone cement was questionable because of relatively few hips with bone cement.

### Strengths

The main strengths of the study were that the CE angle at skeletal maturity was available in the great majority of patients and that information concerning THA could be obtained in all the patients because of the national THA register.

### Conclusions

A previous acetabular shelf operation in patients with persistent DDH does not appear to delay age at THA but seems to prolong the survival of the THA. In patients without previous pelvic surgery, the age at THA is lower in hips with persistent acetabular dysplasia.

*In perspective,* although SA during the last decades for the most part has been replaced by PAO [4,5], recent studies have documented similar long-term outcomes [7,17,18,26,27]. SA is technically a simpler procedure and is associated with less severe complications compared with PAO [27]. Thus, the modified Spitzy procedure could still have a place in the treatment of older children and young adults with moderate degrees of residual acetabular dysplasia and pain, but no hip OA.

## Notes

TT initiated and planned the study, analyzed the data, and wrote the manuscript. SH contributed to planning and performing the study and revision of the manuscript.

The authors would like to thank NAR and especially Anne Marie Fenstad for access to the THA data. They also thank the photographer Øystein Horgmo for help with the illustrations and the statistician Are H. Pripp for help with the statistics.

Handling co-editor: Ilkka Helenius

*Acta* thanks Harald Brismar, Peter John Cundy, Klaus Dieter Parsch, and Joni Serlo for help with peer review of this manuscript.
